# Intestinal Tr1 Cells Confer Protection against Colitis in the Absence of Foxp3^+^ Regulatory T Cell–Derived IL-10

**DOI:** 10.4049/immunohorizons.2200071

**Published:** 2023-06-14

**Authors:** Julie Y. Zhou, Leandre M. Glendenning, Jill M. Cavanaugh, Sarah K. McNeer, Wendy A. Goodman, Brian A. Cobb

**Affiliations:** Department of Pathology, Case Western Reserve University School of Medicine, Cleveland, OH; Department of Pathology, Case Western Reserve University School of Medicine, Cleveland, OH; Department of Pathology, Case Western Reserve University School of Medicine, Cleveland, OH; Department of Pathology, Case Western Reserve University School of Medicine, Cleveland, OH; Department of Pathology, Case Western Reserve University School of Medicine, Cleveland, OH; Department of Pathology, Case Western Reserve University School of Medicine, Cleveland, OH

## Abstract

The intestinal mucosa is continually exposed to diverse microbial and dietary Ags, requiring coordinated efforts by specialized populations of regulatory T cells (Tregs) to maintain homeostasis. Suppressive mechanisms used by intestinal Tregs include the secretion of anti-inflammatory cytokines such as IL-10 and TGF-β. Defects in IL-10 signaling are associated with severe infantile enterocolitis in humans, and mice deficient in IL-10 or its receptors develop spontaneous colitis. To determine the requirement of Foxp3^+^ Treg-specific IL-10 for protection against colitis, we generated Foxp3-specific IL-10 knockout (KO) mice (IL-10 conditional KO [cKO] mice). Colonic Foxp3^+^ Tregs isolated from IL-10cKO mice showed impaired ex vivo suppressive function, although IL-10cKO mice maintained normal body weights and developed only mild inflammation over 30 wk of age (in contrast to severe colitis in global IL-10KO mice). Protection from colitis in IL-10cKO mice was associated with an expanded population of IL-10–producing type 1 Tregs (Tr1, CD4^+^Foxp3^−^) in the colonic lamina propria that produced more IL-10 on a per-cell basis compared with wild-type intestinal Tr1 cells. Collectively, our findings reveal a role for Tr1 cells in the gut, as they expand to fill a tolerogenic niche in conditions of suboptimal Foxp3^+^ Treg-mediated suppression and provide functional protection against experimental colitis.

## Introduction

CD4^+^CD25^+^ regulatory T cells (Tregs) are critical for suppressing immune responses, and defects in Treg frequency or function are associated with chronic inflammation and autoimmunity. The maintenance of immune tolerance is especially important at mucosal barrier sites such as the intestine, which are continually exposed to environmental Ags and commensal microbes. Intestinal Tregs are phenotypically and functionally heterogeneous and use complementary strategies to maintain immune homeostasis in the gut (1). One such mechanism is the production of IL-10, a key anti-inflammatory cytokine originally reported to suppress activation and effector functions of Th1 cells (2). Since its initial discovery, IL-10 has been found to exert numerous functions aimed at maintaining immune homeostasis and reducing host tissue damage during the resolution of inflammation. These include downregulation of Th1-associated cytokine responses and inhibition of activated macrophage responses, including cytokine production, NO synthesis, and Ag presentation (3).

The potent suppressive function of IL-10 is illustrated by the development of very early onset inflammatory bowel disease (VEO-IBD), defined as presentation of IBD in children <6 y of age (4), among patients with monogenic variants in IL-10 or its receptors (5, 6). Most monogenic VEO-IBD cases occur in children <2 y of age (infantile-onset IBD), with extremely high prevalence in the first 6 mo of age (6). Infantile-onset IBD is associated with severe symptoms including colitis, high fevers, and failure to thrive. Although many causative monogenic variants have been identified among VEO-IBD patients, defects in IL-10 signaling are frequently life-threatening (7).

The essential role for IL-10 in maintaining intestinal homeostasis is highlighted by mice lacking global expression of IL-10 (IL-10 knockout [KO]) (8) or its receptor, IL-10Rb (9). These mice develop spontaneous colitis that replicates many features of human IBD, including impaired epithelial barrier function, epithelial hyperplasia, crypt abscesses, ulceration, and infiltration of inflammatory cells to the lamina propria and submucosa (10, 11). Development of colitis in IL-10KO mice is driven by expansion of Th1 and Th17 cells and has historically been attributed to the loss of IL-10 derived from Foxp3^+^ intestinal Tregs (12). However, in previous work, Foxp3-specific deletion of IL-10 resulted in only modest colitis compared with the severe colitis observed in IL-10KO mice (12). Thus, the relative contribution of Foxp3^+^ Treg-intrinsic IL-10 versus IL-10 derived from other cellular sources is unclear.

In addition to naturally occurring Foxp3^+^ Tregs, several other cell types are capable of producing IL-10 and may confer protection in the absence of Foxp3-specific IL-10. These include macrophages (13), follicular T cells (14, 15), regulatory B cells (16), and CD4^+^Foxp3^−^ Tr1 Tregs (17, 18), which are highly enriched in the gut (19), where they differentiate from naive T cells in the presence of IL-10 (20). Interestingly, intestinal Tr1 cells can also be derived from Th17 cells (21), suggesting that Tr1 cells can inhibit pathogenic T cells not only through cytokine-mediated suppression, but also by co-opting their identity. Recent work has revealed unique immunoprotective functions for intestinal Tr1 cells, including suppression of effector T cell proliferation and myeloid cell activation, as well as secretion of IL-22 to promote barrier function (22). These are critical for both maintenance of homeostasis, as well as protection against IBDs (17, 22, 23).

Based on previous studies showing only mild colitis in mice lacking Foxp3^+^ Treg-specific IL-10 (12), together with newly appreciated insights regarding intestinal Tr1 cells (22), we hypothesized that intestinal Tr1 cells may provide adequate immunosuppression to limit inflammation in experimental colitis models. In this study, we assessed the role of intestinal Tr1 cells in protecting against experimental colitis by using the IL-10KO model. We found that mice lacking IL-10 expression specifically in Foxp3^+^ cells (IL-10 conditional KO [cKO]) (24) maintained near normal body weight over time and developed only mild colonic inflammation compared with global IL-10KO mice, despite impaired suppressive function of Foxp3^+^ Tregs in ex vivo assays. We evaluated the frequencies and phenotypes of Foxp3^+^ Tregs and Foxp3^−^ Tr1 cells in IL-10cKO mice and found that CD4^+^Foxp3^−^ Tr1 cells were significantly more abundant in the colonic lamina propria of IL-10cKO mice compared with wild-type (WT) controls. Colonic Tr1 cells isolated from IL-10cKO mice expressed significantly more IL-10 compared with cells isolated from WT mice, effectively compensating for the lack of Foxp3^+^ Treg-derived IL-10. These findings suggest that intestinal Tr1 cells are a highly dynamic population capable of expanding in response to impaired Foxp3^+^ Tregs, thus providing essential protection against experimental colitis.

## Materials and Methods

### Mice

C57BL/6J (stock no. 000664), *Il10^GFP^* (B6.129S6-Il10*^tm1Flv^*/RthsnJ, stock no. 008379), *Foxp3^RFP^* (C57BL/6-*Foxp3^tm1Fl^*^v^/J, stock no. 008374), *Foxp3^creYFP^* (B6.129(Cg)-*Foxp3^tm4(YFP/icre)Ayr^*/J, stock no. 016959), and *Il10*^−/−^ (B6.129P2-*Il10^tm1Cgn^*/J, stock no. 002251) mice, all on the C57BL/6 background, were purchased from The Jackson Laboratory (Bar Harbor, ME). *Il10^fl/fl^* mice were a gift from Dr. Werner Muller (25) via Dr. Asma Nusrat (26). *Foxp3^RF^*^P^ and *Il10^GFP^* mice were crossed to produce *Il10^GFP^* × *Foxp3^RFP^* dual-reporter animals, and *Il10^fl/fl^* and *Foxp3^cre-YFP^* were crossed to generate Foxp3-specific IL-10KO (IL-10cKO) mice in our facility as previously described (24). Mice were housed in a specific pathogen-free facility with a 12-h light/12-h dark cycle. Mice were fed a standard diet (Purina 5010). Mouse housing and studies were approved by and performed according to the guidelines established by the Institutional Animal Care and Use Committee at Case Western Reserve University.

### Cell purification

Primary splenocytes and mesenteric lymph node cells were isolated from mice and reduced to a single-cell suspension by being passed first through a sterile 70-µm nylon mesh cell strainer (Fisher Scientific, Hampton, NH) and then a 40-µm nylon mesh cell strainer. Lamina propria mononuclear cells were isolated by sequential digestion in EDTA (to dislodge intestinal epithelial cells) followed by Liberase TL (thermolysin low) (Roche, Basel, Switzerland). CD4^+^ cells were purified by labeling with anti-mouse CD4 (L3T4) followed by magnetic separation using LS columns (Miltenyi Biotec, Bergisch Gladbach, Germany) according to the manufacturer’s protocols. To sort for splenic Foxp3^+^ Tregs and Foxp3^−^ T cells, magnetic bead–selected CD4^+^ cells were sorted by endogenously expressed Foxp3^RFP^ or Foxp3^YFP^ on a BD FACSAria SORP (special order research product) flow cytometer (BD Biosciences, Franklin Lakes, NJ).

### Cell culture

CD4^+^ cells were cultured in 96-well round-bottom plates (Corning, Corning, NY) at 50,000 cells per well in advanced RPMI 1640 (Life Technologies/Fisher Scientific, Waltham, MA) supplemented with 5% Australian-produced heat-inactivated FBS, 55 µM 2-ME, 100 U/ml and 100 µg/ml penicillin/streptomycin, and 0.2 mM l-glutamine (Life Technologies/Fisher Scientific, Waltham, MA) at 5% CO_2_, 37°C. For CD4^+^ activating conditions, wells were coated with anti-CD3ε (eBioscience, San Diego, CA) at 2.5 µg/ml in PBS, incubated at 4°C overnight, and washed twice with PBS before receiving cells. Where indicated, cultures were supplemented with rIL-2 (R&D Systems, Minneapolis, MN) with and without IL-4 (R&D Systems, Minneapolis, MN), with the equimolar final concentration being 728 pM.

### T cell suppression assay

Tregs were prestimulated with anti-CD3ε, IL-2, and IL-4 for 3 d, washed, and then cultured alone or together on anti-CD3–coated plates with 50,000 freshly isolated and CellTrace-stained WT conventional T (Tconv) cells at a 1:2, 1:4, 1:8, 1:16, 1:32, and 1:64 Treg/Tconv cell ratio (27). Tregs were counted after stimulation and before placing in coculture. The percent suppression was calculated by the CellTrace signal in Tconv cells detected by flow cytometry after 3 d of coculture.

### Flow cytometry

Cells were incubated with UV live/dead stain (Thermo Fisher Scientific, Waltham, MA) and TruStain FcX block (BioLegend, San Diego, CA) prior to Ab staining. IL-10 detection was performed using the IL-10 secretion assay following overnight ex vivo stimulation (Miltenyi Biotec). Cells were stained with Abs to CD4-allophycocyanin Fire 780 (BioLegend), T cell Ig and mucin domain containing 3 (Tim3)-allophycocyanin (BioLegend), CTLA4-allophycocyanin (BioLegend), CD49b-AF647 (BioLegend), CD49b-PE-Cy7 (BD Biosciences), programmed cell death protein 1 (PD1)–allophycocyanin (BioLegend), PD1-BV421 (BioLegend), lymphocyte activating gene 3 (Lag3)–BV785 (BioLegend), F4/80-BV711 (BD Biosciences), and Foxp3-AF488 (BioLegend). Cells were washed in MACS buffer before being analyzed on either a FACSAria SORP (BD Biosciences), LSRFortessa (BD Biosciences), or Attune NxT (Thermo Fisher Scientific) with support of the Cytometry & Imaging Microscopy Core Facility of the Case Comprehensive Cancer Center. Cell sorting was performed on a FACSAria SORP (BD Biosciences). All FACS data were analyzed using FlowJo (Tree Star, Ashland, OR).

### Luminex

Media from Foxp3^+^ Treg cultures and Foxp3^−^ Tconv cell cultures were snap-frozen in liquid nitrogen and shipped to Eve Technologies (Calgary, ON, Canada) for mouse 31-plex cytokine/chemokine analysis and three-plex TGF-β analysis.

### Histology and microscopy

Freshly harvested colon samples were flushed with PBS to remove fecal matter, cut longitudinally, and fixed in Bouin’s solution for 24 h prior to paraffin embedding, sectioning, and staining by the Digestive Disease Research Core Center at Case Western Reserve University. Images were acquired using an Olympus VS120 slide scanner equipped with a ×10 objective and ⅔-inch high-sensitivity/high-resolution charge-coupled device camera (Olympus Life Science, Waltham, MA). All other harvested tissues were fixed in 10% formalin (VWR, Radnor, PA) for 24 h and sent to AML Laboratories (Jacksonville, FL) for paraffin embedding and sectioning. Sectioned slides were stained with H&E in our laboratory. Images were acquired using a high-speed microscope camera (AmScope, Irvine, CA) on a Leica TCS SP5 confocal microscope (Leica, Wetzlar, Germany).

### Data analysis

All data are represented as mean ± SEM. Data and statistical measurements were generated with GraphPad Prism (v9.0). For comparisons between two groups, a Student *t* test was used; comparisons between multiple groups used a two-way or three-way ANOVA.

## Results

### Foxp3-specific IL-10KO mice maintain body weight over 30 wk of age

**FIGURE 1.**
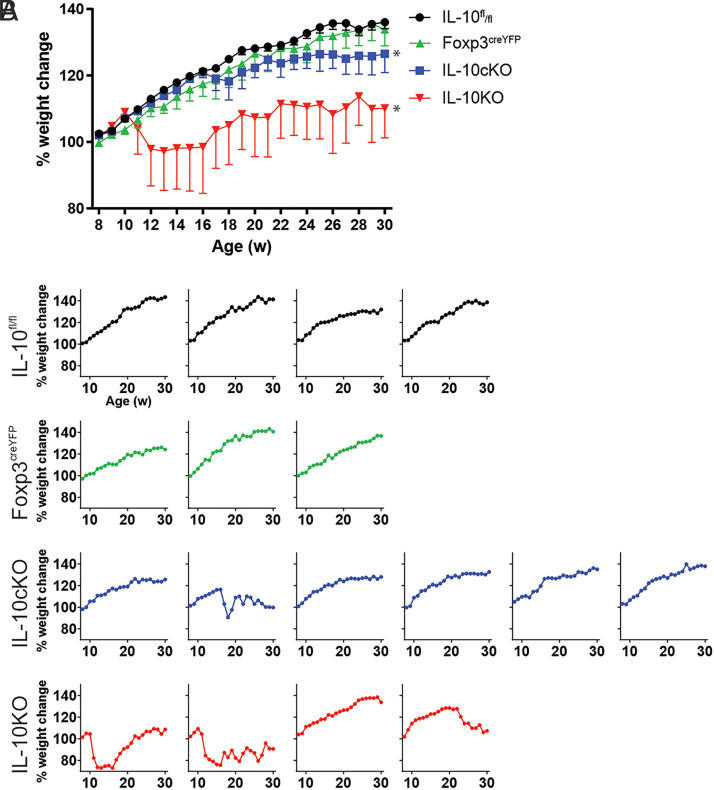
IL-10cKO mice are protected from spontaneous weight loss. Weekly body weights were recorded for IL-10^fl/fl^, Foxp3^cre/YFP^, IL-10cKO, and global IL-10KO mice between 8 and 30 wk of age (*n* = 3–6 mice/group). Percent change was calculated for each animal by dividing weekly weight by initial weight at week 8. (**A** and **B**) Average change for (A) each group and (B) each animal are shown.

Global IL-10KO mice are a well-established model of colitis, typically developing spontaneous disease by 12 wk of age (8). To better understand the role of Foxp3-specific IL-10 in protecting against colitis, we tracked changes in body weight among Foxp3-specific IL-10cKO mice (24) during a 30-wk time course. As expected, control mice (IL-10^flox/flox^ and Foxp3^cre/YFP^) steadily gained weight over time, whereas global IL-10KO mice did not (Fig. 1A, Supplemental Fig. 1A). Interestingly, weight gain among IL-10cKO mice (blue line, Fig. 1A) was comparable to that of control cohorts, with significant differences only observed at weeks 26, 27, and 29 (Supplemental Fig. 1A). To better understand the contributions of each IL-10cKO mouse to the weight changes observed in Fig. 1A, we analyzed the weight of each individual mouse over time. We found that all but one IL-10cKO mouse retained a healthy weight trajectory indistinguishable from that of WT mice, whereas most IL-10KO mice showed significant weight loss (Fig. 1B). These data suggest that IL-10cKO mice have a significantly lower penetrance of wasting disease compared with global IL-10KO mice.

### Foxp3-specific IL-10KO mice are protected from early gastrointestinal-associated inflammatory pathologies

**FIGURE 2.**
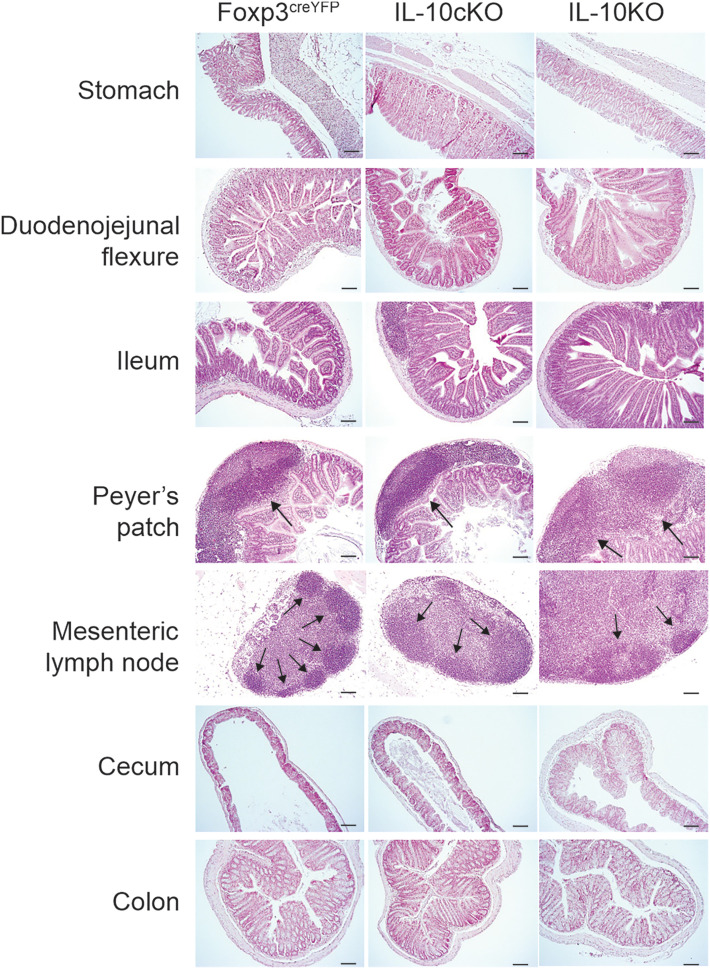
IL-10cKO mice exhibit mild histologic inflammation in the gastrointestinal tract. Ten-week-old mice were euthanized, and gastrointestinal tissues were formalin fixed and paraffin embedded. Representative H&E images are shown from the stomach, duodenojejunal flexure, ileum, Peyer’s patches, mLNs, cecum, and colon (original magnification, ×10; scale bars, 100 μm; results are representative of three total samples).

To understand the impact of IL-10 ablation in Foxp3^+^ cells and to expand on previous studies (12), we performed a thorough necropsy with histologic analysis of animals at 10 wk of age, prior to the onset of significant weight loss in IL-10KO mice (Fig. 1). No substantial findings were noted upon necropsy and gross inspection of the mice (data not shown). H&E staining of the gastrointestinal tract included the stomach, duodenojejunal flexure, ileum, Peyer’s patches, mesenteric lymph nodes (mLNs), cecum, and colon (Fig. 2). Despite the lack of weight loss at this time point, histologic analyses revealed inflammatory changes in IL-10KO mice compared with Foxp3^cre/YFP^ (FP3^creYFP^, control) and IL-10cKO cohorts. The most profound changes included drastic enlargement and disorganization of germinal centers in the mLNs and Peyer’s patches of IL-10KO mice. Additional changes were seen in the cecum, where IL-10KO mice showed profound hypertrophy of the single epithelial cell layer, and IL-10cKO mice showed an intermediate phenotype (Fig. 2). H&E histology of additional tissues, including lung, heart, liver, spleen, kidney, thymus, pancreas, brain, skeletal muscle, skin, and adipose tissue were analyzed with no histological changes noted (Supplemental Fig. 1B).

### Foxp3-specific IL-10KO mice are protected from severe colitis

**FIGURE 3.**
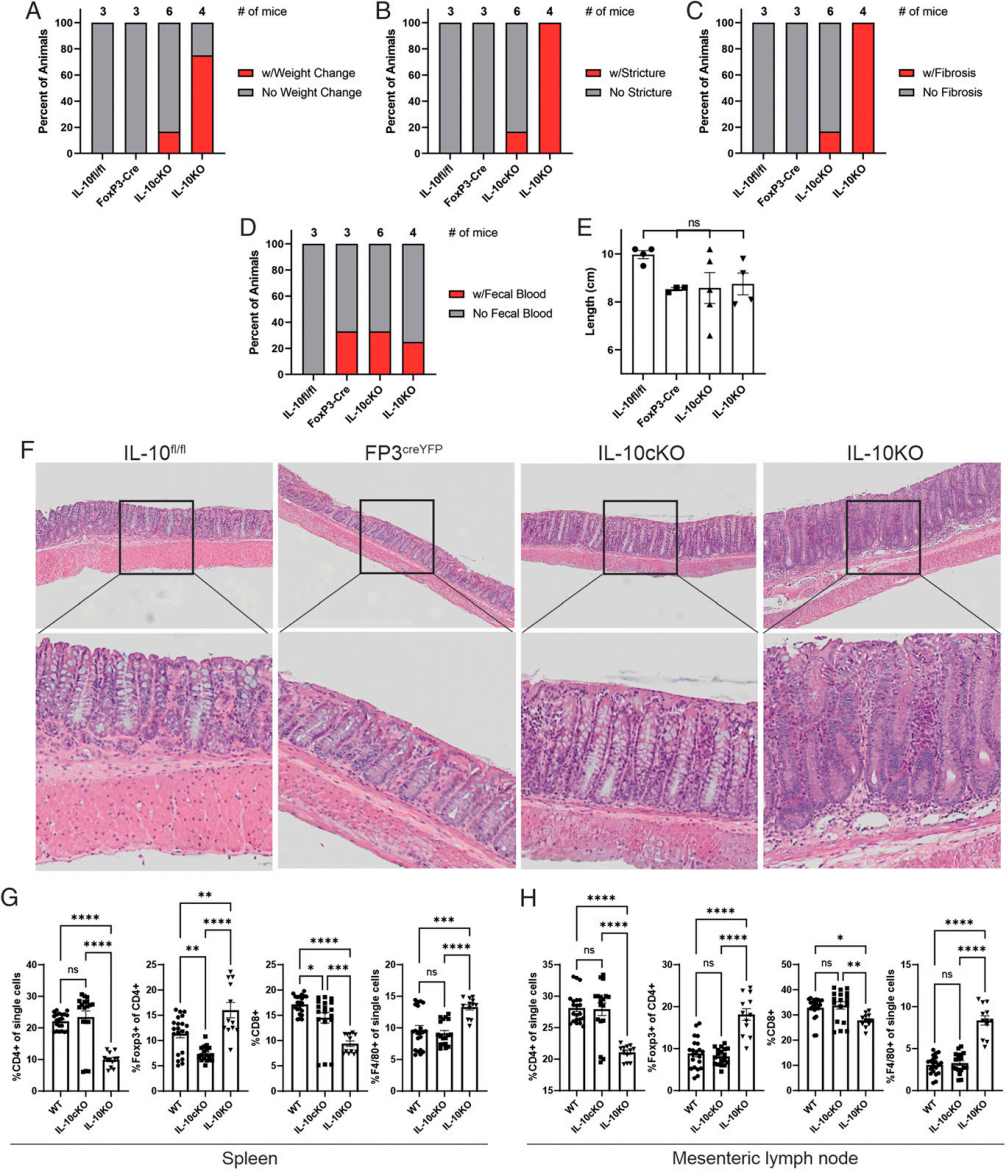
IL-10cKO mice are protected from severe colitis. Experimental colitis was assessed in IL-10^fl/fl^, Foxp3^cre/YFP^, IL-10cKO, and global IL-10KO mice at 30 wk of age. (**A**–**D**) Percentages of mice exhibiting (A) weight loss ≥20%, (B) colonic stricturing, (C) colonic fibrosis, and (D) fecal blood within each cohort are shown (data represent percent of mice exhibiting each symptom; *n* = 3–6/group). (**E**) Colon lengths were measured at the time of harvest (data represent mean ± SEM; *n* = 3–5 mice/group). (**F**) Representative H&E-stained colon tissues (top: ×10 with 3.3-fold original magnification; bottom: ×10 with 10-fold original magnification). (**G** and **H**) Flow cytometric analysis of single-cell suspensions from (G) spleen and (H) mesenteric lymph nodes show percentages of CD4^+^, CD8^+^, F4/80^+^, and CD4^+^Foxp3^+^ cells within each sample (data represent mean ± SEM; *p ≤ 0.05, **p ≤ 0.01, ***p ≤ 0.001, ****p ≤ 0.0001; ns, not significant; *n* = 3–6, with technical triplicates shown/group).

Experimental colitis was assessed in IL-10cKO mice at 30 wk of age and compared with that of global IL-10KO mice and WT controls. As expected, IL-10KO mice exhibited significant weight loss (Fig. 3A) and increased frequency of colonic stricturing (Fig. 3B), fibrosis (Fig. 3C), and fecal blood (Fig. 3D) compared with IL-10^fl/fl^ and Foxp3^cre/YFP^ controls. All IL-10KO mice presented with at least one of the measures of disease, indicating disease penetrance of 100%. In contrast, IL-10cKO mice exhibited low disease penetrance, with only one animal exhibiting overt disease as assessed by weight loss and colonic stricturing/fibrosis (Fig. 3A–C). Although colon lengths (Fig. 3E) were comparable among all cohorts, gross colon morphology revealed significant hypertrophy and stricturing among IL-10KO mice that was only observed in one IL-10cKO mouse (Supplemental Fig. 1C, mouse 9). This suggests that although most IL-10cKO mice are spared from severe colitis, overt disease can develop in selected individuals.

Next, we examined H&E-stained longitudinal colon tissues from IL-10cKO mice and controls (30 wk of age) to characterize histological inflammation. Compared to control IL-10^fl/fl^ and Foxp3^cre/YFP^ tissues, colons of IL-10KO mice showed significant hypertrophy, thickening of the muscularis mucosae, goblet cell depletion, crypt abscesses, and increased mononuclear cell infiltrates to the lamina propria and submucosa (Fig. 3F). In contrast, colon tissue from the most representative IL-10cKO mouse (median of the group for colitis parameters in Fig. 3A–D) showed none of these histologic findings and was indistinguishable from the uninflamed IL-10^fl/fl^ and Foxp3^cre/YFP^ samples (Fig. 3F).

To determine whether there are cellular changes outside of the colon, we used either YFP reporter fluorescence (WT, IL-10cKO) or Foxp3 staining (IL-10KO) and flow cytometry to immunophenotype the spleen (Fig. 3G) and mLNs (Fig. 3H) of IL-10cKO mice and controls. Foxp3 detection by Ab and reporter detection was validated to ensure comparable findings (Supplemental Fig. 2A, 2B). We found that although IL-10KO mice had significant reductions in the proportion of CD4^+^ T cells, increased frequencies of Foxp3^+^ Tregs, and decreased F4/80^+^ macrophages (Fig. 3E, 3F), IL-10cKO did not exhibit these changes and, in most parameters, appeared indistinguishable from WT controls.

### Phenotypic and functional alterations in IL-10–deficient Foxp3^+^ Tregs

**FIGURE 4.**
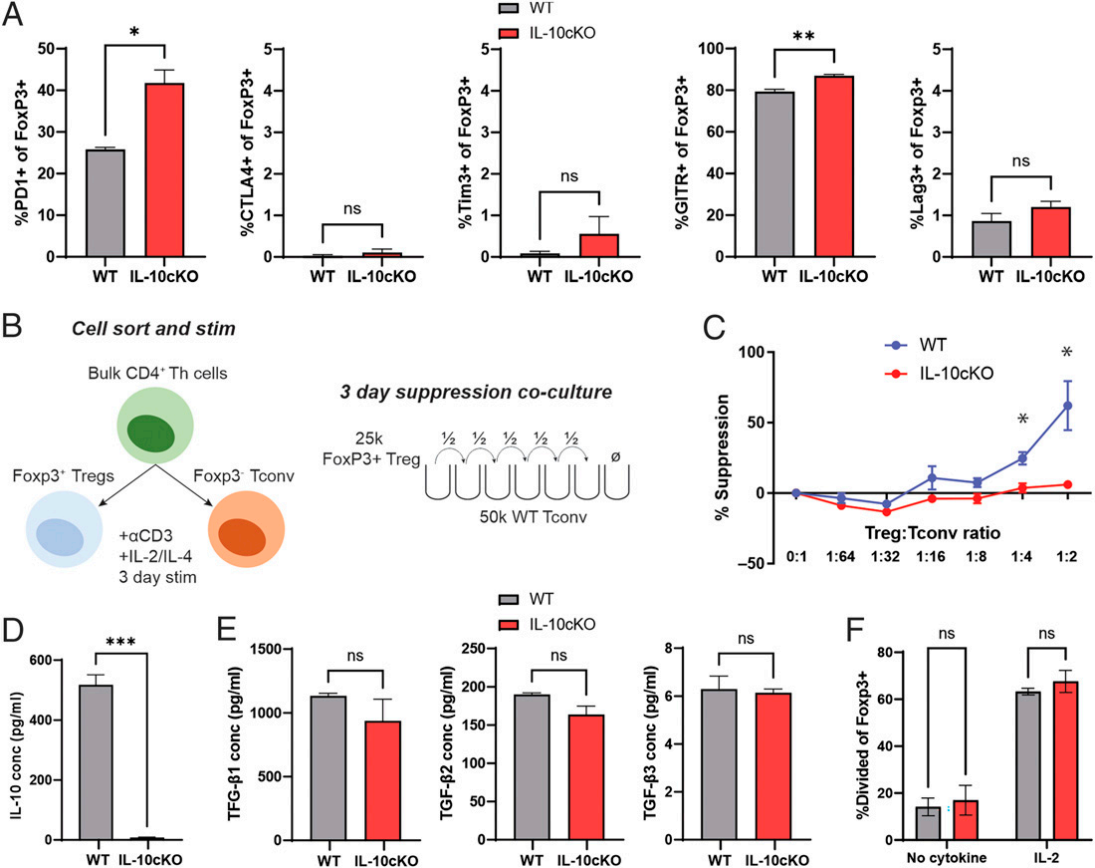
IL-10–deficient Foxp3^+^ Tregs in IL-10cKO mice lack optimal suppressive function. (**A**) Foxp3^+^ Tregs were purified and activated on anti-CD3–coated plates with IL-2 supplementation for 3 d, after which surface expression of inhibitory receptors was assessed by flow cytometry (data represent mean ± SEM, *n* = 3/group). (**B**) Schematic of the Treg suppression assay. (**C**) Freshly isolated CD4^+^Foxp3^−^ Tconv cells were labeled with CellTrace and cocultured with anti-CD3 and the indicated ratios of Tregs. Tconv cell proliferation and Treg suppression were determined by dye dilution after 3 d of coculture (data represents mean ± SEM, *n* = 3/group). (**D** and **E**) Supernatants were collected from Foxp3^+^ Tregs following 3 d of stimulation on anti-CD3–coated plates with IL-4 and analyzed for (D) IL-10 and (E) TGF-β1–TGF-β3. (**F**) Foxp3^+^ Tregs were purified, labeled with CellTrace, stimulated on anti-CD3–coated plates for 3 d, and assessed for proliferation by flow cytometry. Data represent mean ± SEM. *p ≤ 0.05, **p ≤ 0.01, ***p ≤ 0.001; ns, not significant; *n* = 3/group.

Because IL-10cKO mice did not show high prevalence of severe colitis (Figs. 1–3), we asked whether Foxp3^+^ Tregs in these mice may compensate for the loss of IL-10 by upregulating other suppressive mechanisms to maintain homeostasis. First, we examined expression of well-known inhibitory receptors on Foxp3^+^ cells isolated from WT versus IL-10cKO mice (Fig. 4A). CD4^+^Foxp3^+^ Tregs were isolated by flow cytometric sorting, cultured on anti-CD3–coated plates for 3 d, and then stained for expression of PD1, CTLA4, Tim3, glucocorticoid-induced TNFR-related protein (GITR), and Lag3 by flow cytometry. PD1 and GITR were strongly expressed by Foxp3^+^ Tregs, and their expression was significantly enhanced in cells isolated from IL-10cKO mice compared with controls (Fig. 4A, Supplemental Fig. 2C). In contrast, CTLA4, CD49b, and Lag3 were lowly expressed and not significantly enhanced in IL-10cKO Foxp3^+^ Tregs (Fig. 4A, Supplemental Fig. 3A).

We next tested the suppressive function of IL-10cKO Tregs using a standard ex vivo assay (28). CD4^+^Foxp3^+^ Tregs were isolated by flow sorting, activated using plate-bound anti-CD3, IL-2, and IL-4 (24), and then cocultured together with freshly isolated, CellTrace-labeled Tconv cells (CD4^+^Foxp3^−^, Fig. 4B). Suppression of Tconv cell proliferation was assessed by flow cytometry following 3 d of coculture. Despite increased expression of PD1 and GITR, IL-10cKO Tregs showed significantly impaired suppression compared with WT Tregs at high Treg/Tconv cell ratios (Fig. 4C, Supplemental Fig. 3B).

Next, we assessed the protein production of 32 cytokines and chemokines by Foxp3^+^ Tregs using a Luminex multiplex analysis. Other than IL-10 itself, which was undetectable in IL-10cKO Tregs (Fig. 4D), no soluble factors were differentially expressed by IL-10cKO versus WT Tregs (Supplemental Fig. 2C). Because Foxp3^+^ Tregs are known to secrete large quantities of the suppressive cytokine TGF-β, we tested the production of TGF-β1, TGF-β2, and TGF-β3 and found equivalent expression of these three factors among IL-10cKO and WT Tregs (Fig. 4E).

The proliferation of Foxp3^+^ Tregs can be driven by many factors including their surrounding cytokine milieu (24). We therefore asked whether the absence of secreted IL-10 and its associated autocrine signaling may impact Foxp3^+^ Treg proliferation in IL-10cKO mice. CD4^+^Foxp3^+^ Tregs were purified, labeled with CellTrace, cultured for 3 d on anti-CD3–coated plates, and assessed for proliferation. Proliferation of IL-10cKO and WT Tregs was equivalent (Fig. 4F), suggesting that IL-10 is dispensable for Foxp3^+^ Treg proliferation ex vivo. Collectively, these results show that although IL-10–deficient Foxp3^+^ Tregs upregulate expression of PD1 and GITR, they do not have enhanced suppressive function or significant changes to their proliferation or cytokine production.

### Tr1 cells compensate for the lack of IL-10 from Foxp3^+^ Tregs

Most IL-10cKO mice maintained intestinal homeostasis (Figs. 1–3), despite impaired functional suppression and lack of compensatory mechanisms among IL-10–deficient Foxp3^+^ Tregs (Fig. 4). Therefore, we asked whether anti-inflammatory functions of other immune cell populations may be enhanced in IL-10cKO mice, compensating for the lack of Foxp3^+^ Treg-derived IL-10.

**FIGURE 5.**
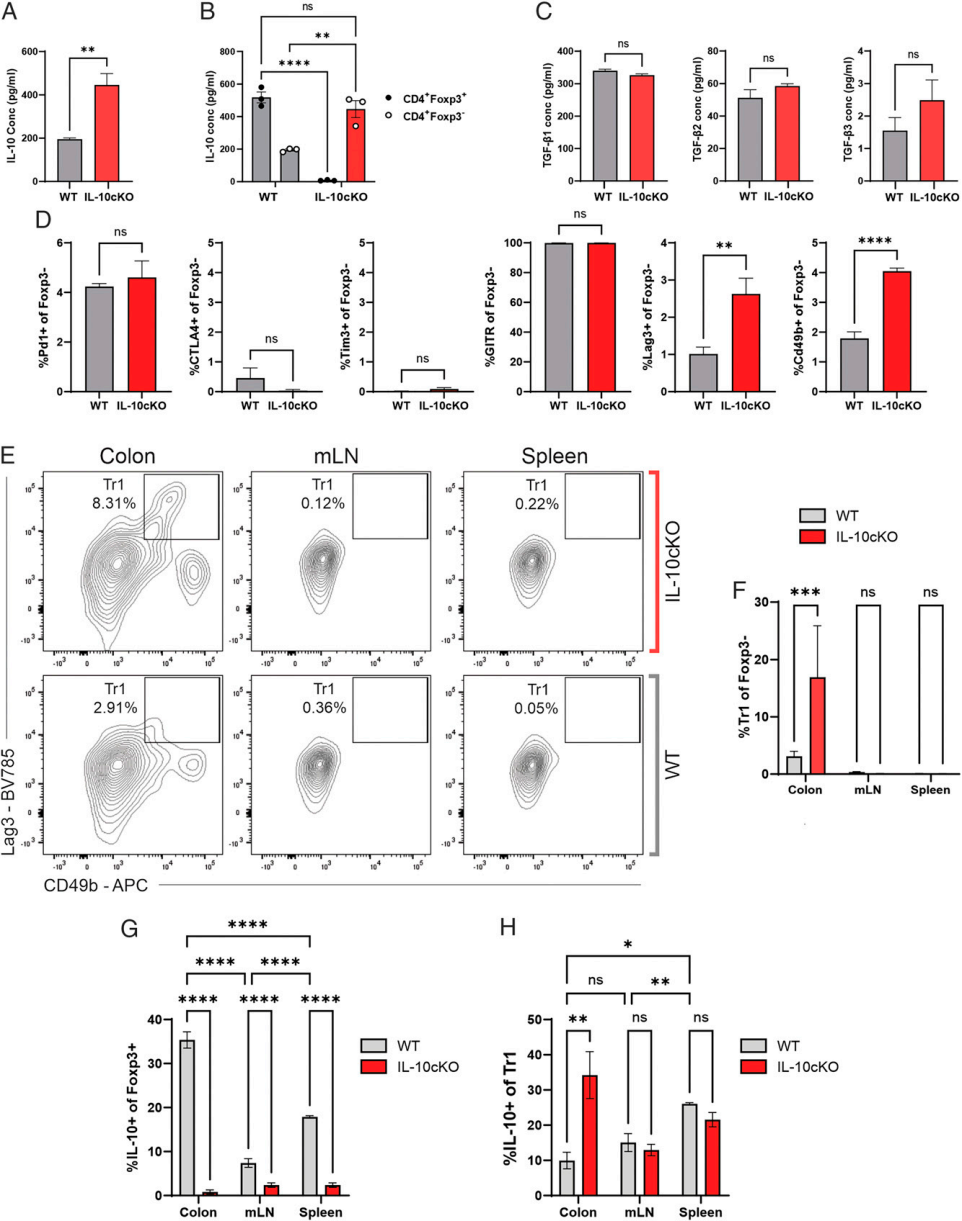
Foxp3^−^ T cells from IL-10cKO mice secrete high levels of IL-10. (**A**) Supernatants from anti-CD3– and IL-2–treated Foxp3^−^ T cells were collected after 3 d of culture and assayed for IL-10 secretion by Luminex. (**B**) Comparison of IL-10 secreted by Foxp3^+^ and Foxp3^−^ T cells. (**C**) Supernatants from anti-CD3– and IL-2–treated Foxp3^−^ T cells were collected after 3 d of culture and assayed for TGF-β1–TGF-β3 secretion by Luminex. (**D**) Foxp3^−^ T cells were purified from mLNs and activated on anti-CD3–coated plates with IL-2 for 3 d, after which surface expression of indicated markers was quantified by flow cytometry. (**E** and **F**) Flow cytometric analysis of Tr1 markers in CD4^+^Foxp3^−^ cells isolated from indicated tissues, expressed as percentages of CD4^+^Foxp3^−^ singlet lymphocytes. (**G** and **H**) The IL-10 capture system and subsequent flow cytometry were used to ascertain IL-10 production by Foxp3^+^ Tregs or Foxp3^−^ Tr1 cells. Data represent mean ± SEM. *p ≤ 0.05, **p ≤ 0.01, ***p ≤ 0.001, ****p ≤ 0.0001; *n* ≤ 3/group.

First, we examined cytokine production by CD4^+^Foxp3^−^ cells after 3 d of culture on anti-CD3–coated plates by Luminex. Strikingly, Foxp3^−^ T cells from IL-10cKO mice produced more than double the amount of IL-10 compared with that of WT Foxp3^−^ T cells (Fig. 5A). In fact, the amount of IL-10 produced by IL-10cKO Foxp3^−^ Tconv cells on a per cell basis was comparable to that produced by Foxp3^+^ Tregs from WT mice (Fig. 5B), strongly suggesting a mechanism by which CD4^+^Foxp3^−^ T cells could compensate for the loss of Foxp3^+^ Treg-specific IL-10. Production of TGF-β1, TGF-β2, and TGF-β3 was unchanged in Foxp3^−^ T cells isolated from IL-10cKO mice compared with WT mice (Fig. 5C), although IL-1a and IL-13 showed modest changes (Supplemental Fig. 4A). All other surveyed cytokines were equivalent between Foxp3^−^ T cells isolated from IL-10cKO and WT mice (Supplemental Fig. 4B).

Because the secretion of IL-10 was significantly elevated in Foxp3^−^ T cells from IL-10cKO mice (Fig. 5A), we evaluated the expression of surface markers associated with tolerogenic T cell function in CD4^+^Foxp3^−^ T cells isolated from IL-10cKO versus WT mice. Although there was no difference in PD1, CTLA4, Tim3, and GITR, expression of Lag3 and CD49b was significantly elevated in Foxp3^−^ T cells from IL-10cKO mice (Fig. 5D). Lag3 and CD49b are classically expressed by Tr1 cells (29), a peripherally induced population of Foxp3^−^ Tregs capable of producing high levels of IL-10 following Ag exposure (17, 18). We assessed the frequency of Lag3^+^CD49b^+^ Tr1 cells in the colonic lamina propria, mLNs, and spleen of IL-10cKO and WT mice by flow cytometry (Fig. 5E), finding that the Tr1 population was significantly expanded in colons of IL-10cKO mice (Fig. 5F). Although Tr1 cells have been reported to be highly prevalent in the small intestine (30), our analysis of IL-10–producing cells revealed the highest proportion of IL-10^+^ Tr1 cells in the colons of IL-10cKO mice (Supplemental Fig. 4C).

To understand whether the increased frequency of Lag3^+^CD49b^+^ Tr1 cells is accompanied by increased IL-10 production, we measured IL-10 secretion among Foxp3^+^ Tregs and Foxp3^−^ Tr1 cells using an IL-10 capture system. In agreement with our Luminex data (Fig. 4D), IL-10 was not secreted by Foxp3^+^ Tregs from IL-10cKO mice (Fig. 5G, Supplemental Fig. 4E). IL-10 secretion among Foxp3^+^ Tregs from WT mice was validated using our previously reported IL-10^GFP^ × Foxp3^RFP^ dual-reporter mouse, which showed significant IL-10 expression among Foxp3^+^ Tregs isolated from the colonic lamina propria and spleen (Supplemental Fig. 4D). Using the IL-10 capture system, we found that colonic CD4^+^Foxp3^−^ Tr1 cells from IL-10cKO mice produce significantly more IL-10 than those from WT mice (Fig. 5H, Supplemental Fig. 4E). Taken together, these data indicate that in conditions of impaired Foxp3^+^ IL-10 production, intestinal Tr1 cells expand in frequency and function to provide functional immune suppression and protect against spontaneous colitis.

## Discussion

Mice with conditional ablation of IL-10 in Foxp3^+^ Tregs (IL-10cKO) are largely spared from the severe colonic disease that global IL-10KO mice develop (Ref. 12 and Figs. 1–3). However, the reason for this remained unclear, especially as we found that Foxp3^+^ Tregs isolated from IL-10cKO mice exhibit impaired suppressive function ex vivo (Fig. 4). In this study, we characterized the CD4^+^Foxp3^+^ and CD4^+^Foxp3^−^ compartments in IL-10cKO mice to better understand how IL-10cKO mice avoid severe colitis, and we identified a critical compensatory mechanism used by intestinal Tr1 cells to confer protection in the absence of Foxp3-specific IL-10.

In this study, we confirmed that IL-10cKO mice have a low prevalence of colitis compared with the extremely high prevalence of severe disease observed in global IL-10KO mice (Figs. 1–3). In response to IL-10 deletion, Foxp3^+^ Tregs upregulated their expression of the contact-dependent inhibitory receptors PD1 and GITR, but the cells were not functionally suppressive in classical ex vivo assays (Fig. 4). This suggests the relative unimportance of Foxp3^+^ Treg-derived IL-10 in this context. Instead of relying solely on Foxp3^+^ Treg suppression to maintain immune homeostasis, we discovered that in IL-10cKO mice, CD4^+^Foxp3^−^ Tr1 cells were expanded in frequency and produced more IL-10 compared with Tr1 cells isolated from WT animals. These data point to a cellular partnership where Tr1 cells can functionally compensate for the lack of Foxp3^+^ cell–derived IL-10, thereby circumventing the onset of colitis caused by the global absence of IL-10.

Although IL-10 is not necessary for the induction of Tr1 cells in vitro (31), IL-10 receptor signaling has been reported as essential for Tr1 cell function and IL-10 production in vivo (32). This explains that although Tr1 cells exist in the global IL-10KO mouse, they are not functionally active and cannot produce IL-10 to compensate for the lack of IL-10 release from other cell types, therefore leading to the development of severe colonic inflammation by 10 wk of age. In contrast, mice conditionally lacking IL-10 only in Foxp3-expressing cells have unperturbed IL-10 production by other immune cell types that secrete the necessary IL-10 to activate Tr1 cells and promote their robust production of the suppressive cytokine. The current lack of a consensus for a Tr1-specific transcription factor increases the difficulty in running confirmatory studies; however, there is promising work showing that the transcription factor BLIMP-1 is associated with Tr1 IL-10 production and may be useful in future studies (33).

Also inherent in this study is the fact that colitis is a complex and heterogeneous disease. Reduced Treg and Tr1 responses have been associated with IBD pathology; however, these changes may not be reflected in every patient (28, 34–36). Interestingly, colitis development in IL-10KO mice is highly dependent on the environment of the local vivarium, indicating a strong role for the host microbiome in development of disease (37, 38). Indeed, IL-10KO colitis does not develop in animals raised in germ-free conditions (39). Microbial byproducts such as short-chain fatty acids have been shown to increase the production of IL-10 by microbe-specific T cells (40), suggesting an important feedback loop between the host microbiome and intestinal Tr1 cells with microbial specificity. Additionally, polysaccharide A derived from *Bacteroides fragilis* has a well-documented ability to suppress multiple rodent models of inflammatory diseases in an IL-10–dependent manner, including asthma, experimental autoimmune encephalomyelitis, and abscess formation (24, 41–43). The mechanism by which it does so involves canonical MHC class II presentation and expanded frequencies of effector memory CD4^+^Foxp3^−^ T cells, which produce IL-2 and IL-4 that efficiently and robustly induce IL-10 expression by Foxp3^+^ Tregs (24, 41, 44–46). Thus, IL-10 signaling is critical for both the induction of intestinal regulatory cells, as well as the function of these cells to suppress inflammation.

In addition to the presence of environmental factors such as commensal microbes, the fluctuation of signaling molecules such as estrogen has been shown to modulate the activity of Foxp3^+^ Tregs to impair gut immune homeostasis (28). These findings strongly point to more complex regulatory circuits involving host-derived factors, such as the microbiome, diet, and hormones, and specialized populations of Tregs including Foxp3^+^ cells and Tr1 cells within the intestinal mucosa. An improved understanding of how these factors influence one another in settings of homeostasis and disease is critical for the eventual design of improved therapeutics for IBD patients.

Collectively, we show that IL-10cKO mice are largely spared from the severe colitis that affects global IL-10KO mice, due to compensation from Tr1 cells in the colonic lamina propria. These data suggest a novel ability for intestinal Tr1 cells to expand in both frequency and function, filling a tolerogenic niche in the absence of Foxp3-derived IL-10 and conferring critical protection from colitis. Overall, our results indicate a regulatory network mediated by cross-talk between Foxp3^+^ and Foxp3^−^ Tregs, wherein Tr1 cells functionally compensate for suboptimal Foxp3^+^ Treg activity.

## Supplementary Material

Supplemental Figures 1 (PDF)
