# Supplementary material for: Intestinal Tr1 Cells Confer Protection against Colitis in the Absence of Foxp3+ Regulatory T Cell–Derived IL-10
Source: Immunohorizons. 2023 Jun 14;7(6):456–66. doi: 10.4049/immunohorizons.2200071 (PMC10580124; doi:10.4049/immunohorizons.2200071)
Supplement: Supplemental Figures 1 (PDF) [file IH_2200071_Supplemental_1.pdf]

Supplemental Figures and Legends

A

Week	IL-10KO vs WT	iL-10cKO vs WT	IL-10KO vs IL-10cKO
8	0.925581853	0.505311648	0.540421479
9	0.027918866	0.713764253	0.169943116
10	0.042271129	0.360294771	0.133067188
11	0.118286951	0.866855863	0.2781764
12	0.005213486	0.711582456	0.068074982
13	0.002898076	0.745630226	0.047067095
14	0.001923676	0.516268111	0.042169579
15	0.001704802	0.961425636	0.033499917
16	0.001072368	0.80042375	0.027281338
17	0.002724543	0.48069579	0.080525913
18	0.001981345	0.185729356	0.164109459
19	0.001817584	0.157964021	0.144485093
20	0.001097906	0.067944826	0.074710239
21	0.001030166	0.284054879	0.053266946
22	0.00121174	0.138414476	0.11256042
23	5.81257E-05	0.110743712	0.025018411
24	1.48097E-05	0.081705274	0.012581151
25	1.95377E-05	0.094146097	0.036953356
26	1.25265E-05	0.039727789	0.016536763
27	1.64205E-05	0.028333115	0.030455572
28	9.78558E-06	0.090774468	0.053811364
29	7.34604E-07	0.045168781	0.023815938
30	3.38169E-06	0.064620612	0.032150482

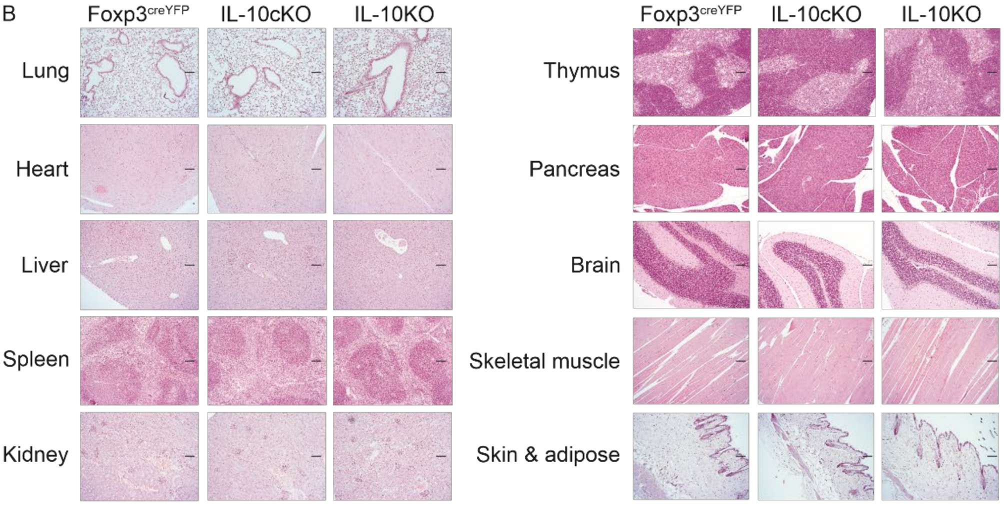

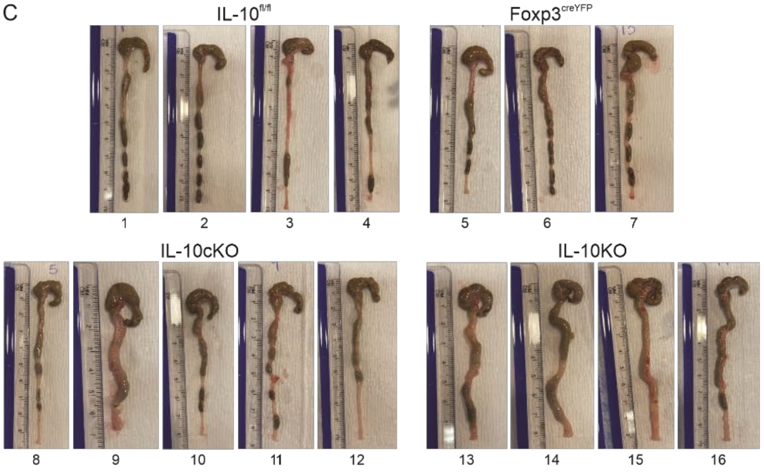

Supplemental Figure S1: Analysis of the conditional knockout
(A) P values comparing the weight loss between WT (IL-10$^{fl/fl}$ and Foxp3$^{cre/YFP}$), IL-10cKO (IL-10$^{fl/fl}$ x Foxp3$^{cre/YFP}$), and IL-10KO mice for each of weeks 8-30. (B) Indicated tissues were obtained from 10-week-old mice, prior to the onset of severe colitis. Representative images are shown from H&E-stained, formalin-fixed, paraffin-embedded tissues (10x magnification with scale bar representing 100 μm). (C) Images of full-length colons obtained from 30-week-old mice.

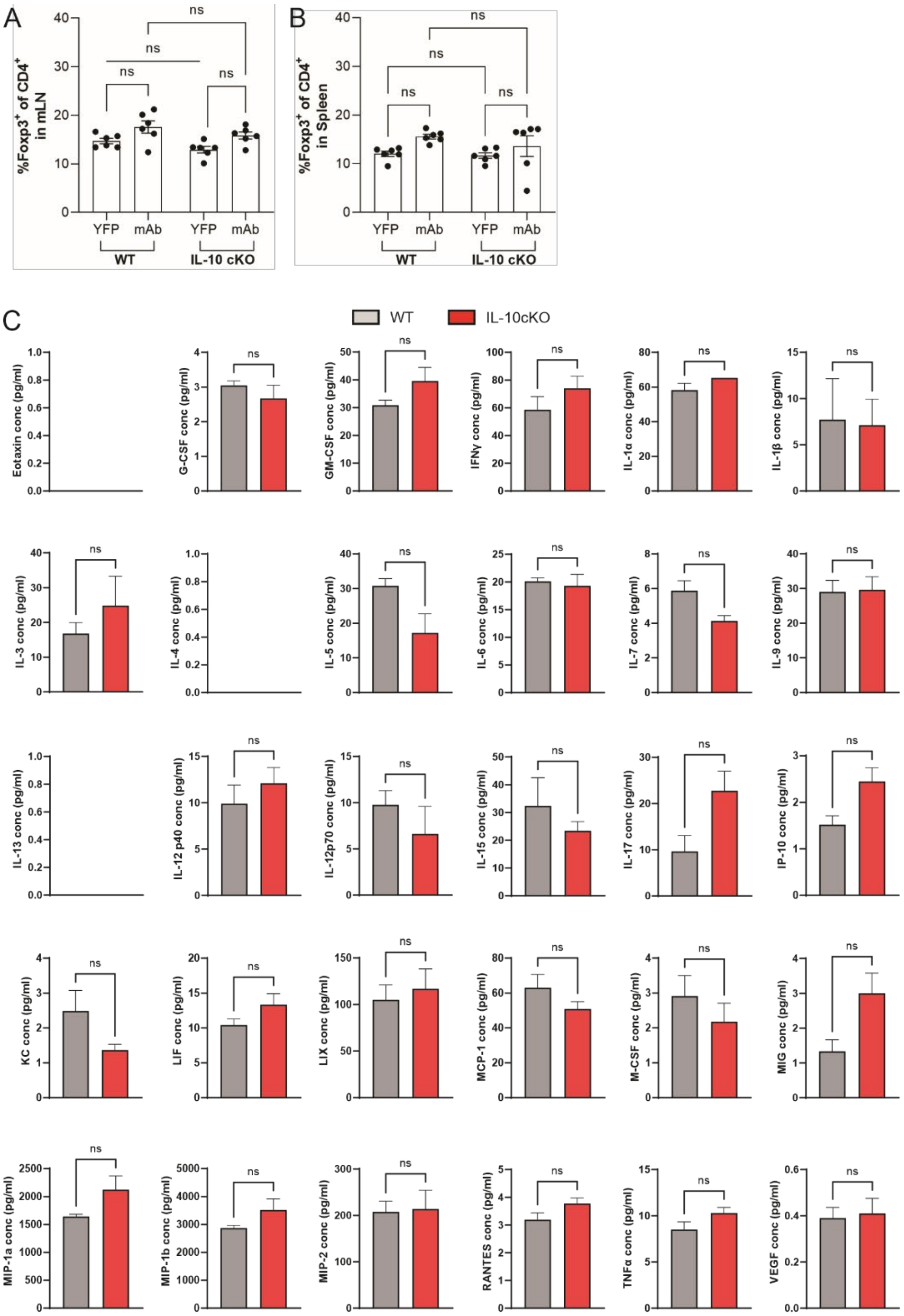

Supplemental Figure S2: Protein expression profiles.
(A-B) CD4 cells were isolated from WT (FP3creYFP) and IL-10cKO mesenteric lymph nodes and spleen. Cells were stained with a Foxp3 antibody and compared to the internal YFP reporter. No difference in the number of Foxp3+ cells for each detection method were present. (C) Foxp3+ Tregs were purified and stimulated on αCD3-coated plates with IL-2 supplementation for 3 days, after which the cytokine content of the supernatants was analyzed by multiplexed Luminex.

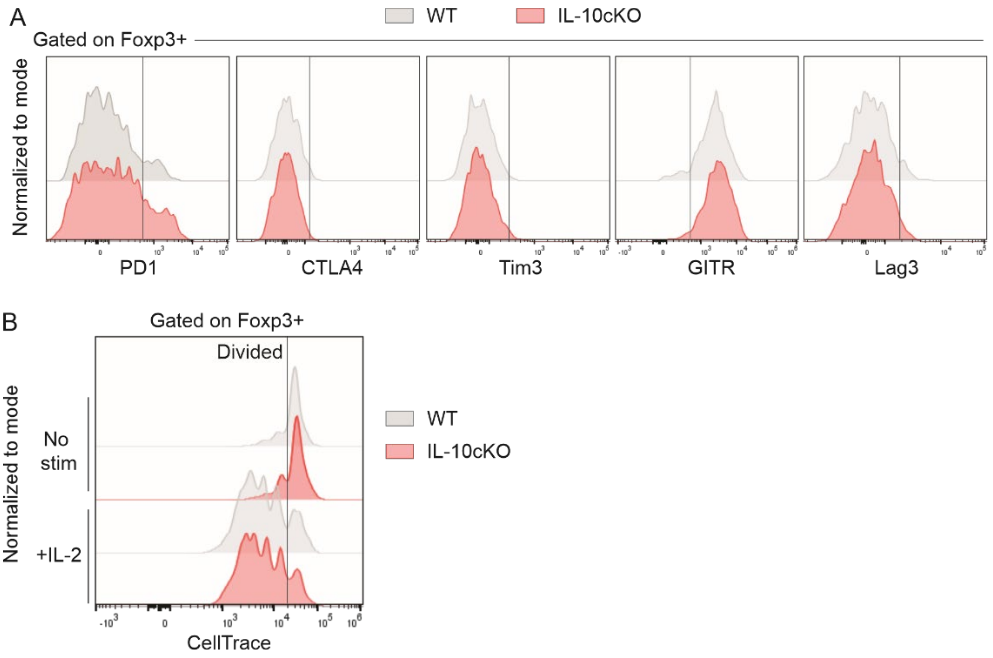

Supplemental Figure S3: Representative flow cytometry histograms for Figure 4
(A) Foxp3+ Tregs were purified and activated on αCD3-coated plates with IL-2 supplementation for 3 days, after which surface expression of inhibitory receptors was assessed by flow cytometry. Quantitation is shown in Figure 4. (B) Representative histograms from Treg suppression assays shown in Figure 4.

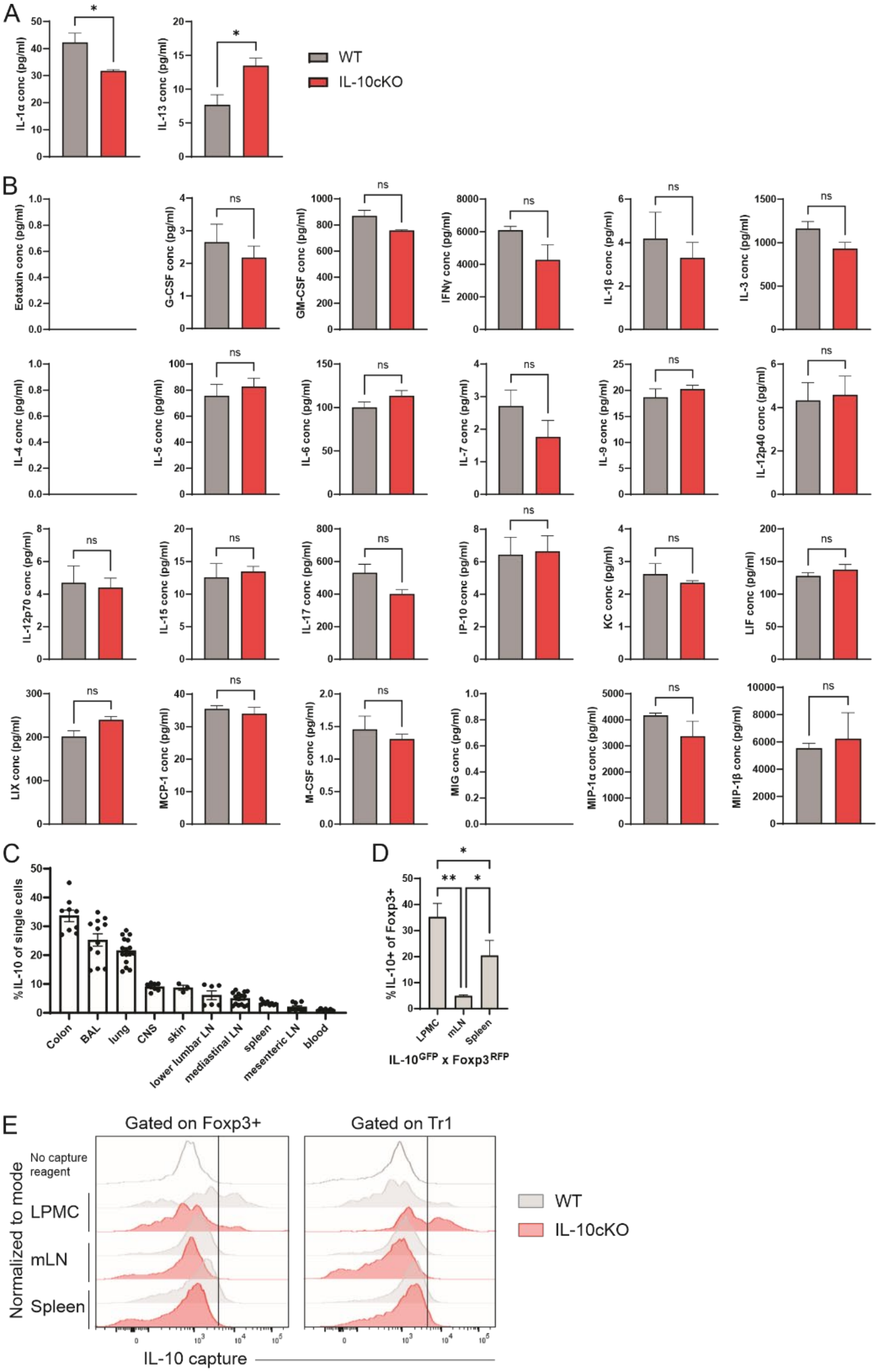

Supplemental Figure S4: Cytokines secreted by regulatory T cells

(A-B) Foxp3^{-} T cells were purified and stimulated on αCD3-coated plates with IL-2 supplementation for 3 days, after which the cytokine content of the supernatants was analyzed by multiplexed Luminex. *(C)* Characterization of IL-10 production across different tissue types by flow cytometry. (D) IL-10 production by Foxp3^{+} Tregs was analyzed by flow cytometry using IL-10^{GFP} x Foxp3^{RFP} dual reporter mice. Data represents mean +/- SEM (NS, not significant; * $p \leq 0.05$, ** $p \leq 0.01$, **** $p \leq 0.001$; n=3/group). (E) Representative histograms from the IL-10 capture system used to ascertain IL-10 production by Foxp3^{+} Tregs and Foxp3^{-} Tr1 cells, as quantified in Figure 5.
